# Editorial: Artificial intelligence and machine learning for drug discovery, design and repurposing: methods and applications

**DOI:** 10.3389/fphar.2023.1333747

**Published:** 2023-11-29

**Authors:** Pan Zheng, Xiangxiang Zeng, Xun Wang, Pingjian Ding

**Affiliations:** ^1^ Information Systems, University of Canterbury, Christchurch, New Zealand; ^2^ Department of Computer Science, Hunan University, Changsha, China; ^3^ College of Computer Science and Technology, China University of Petroleum, Dongying, China; ^4^ Center for Artificial Intelligence in Drug Discovery, School of Medicine, Case Western Reserve University, Cleveland, OH, United States

**Keywords:** artificial intelligence, neural network, drug discovery, drug repurposing, drug design, drug repositioning

## 1 Introduction

With the advancement of artificial intelligence (AI) and machine learning methods, many science and engineering challenges and problems can now be tackled and solved through new computing paradigms. Drug and pharmaceutical research serve as ideal testing grounds for AI and machine learning techniques to realize their full potential. To supply pharmacologically active compounds with a specific function, drug design is crucial to the drug discovery and development process. The idea of Computer-Aided Drug Design was first coined around the 80 s (Kuntz et al., 1982) when the capabilities of both hardware and software were limited. Since the start of the new century, drug discovery, design and repurposing using AI and machine learning approaches have benefited computer-aided pharmaceutical research at all stages of the drug development cycle and attracted the attention of researchers from medical, pharmaceutical, biochemical, computer science and other related fields.

## 2 Drug discovery, design and repurposing

Drug development often includes four phases, namely, drug discovery, pre-clinical research, clinical development (Phase I, II, and III) and the last phase, approval and post-market surveillance (Phase IV) (Tamimi and Ellis, 2009). Drug discovery is the first phase of drug development. It is the process of identifying and developing new chemical compounds that can be used as therapeutic mechanisms to react on the biological targets that are usually protein structures causing various diseases and medical conditions. Many tasks involve the use of computers to simulate, screen, predict and investigate the interactions between the drug and the target, leading to what are known as *in silico* studies. Drug design is commonly regarded as a specific phase within the broader process of drug discovery. It focuses on the design and refinement of potential drug compounds, i.e., lead optimization. Drug repurposing Pushpakom et al (2019) is a technique or process of exploring novel pharmaceutical uses of established medicines that are originally designed for different medical indications. It offers a more cost-effective and faster route to drug development.

## 3 The contributions to the topic

In the following, we summarized the contributions to our Research Topic with a brief background of each.

Computer-aided machine learning methods that work on drug and target data have the potential to reduce both the expenses and time required for Drug-Target Interaction (DTI) and, subsequently, drug development. Previously the efforts of investigating DTI relied on limited source data, often a single source data. Wang et al. present an effective fusion approach using multi-source data of drug and target. The approach, EFMSDTI, builds similarity networks based on multi-source information networks of drugs and targets and fuses them using selective and entropy weighting. The deep neural networks model learns low-dimensional vectors of drugs and targets, and the LightGBM algorithm is used for DTI prediction. Experimental results show that the approach outperforms state-of-the-art algorithms in terms of prediction accuracy.

Studying Protein-protein interactions (PPI) is an important Research Topic of research that delves into drug discovery. Interactions between DNA-related proteins play a vital role in biological processes and the design of drugs. The experimental methods to identify these interactions are expensive and time-consuming. To address this Research Topic, deep learning methods, including graph neural networks, have been developed. Li et al. propose DPB-NBFnet, a deep learning graph neural network model that leverages NBFnets and graph neural networks to predict DNA-protein binding. The experiments of the study are conducted on 100 datasets and the method demonstrates promising performance and provides a computationally efficient solution for predicting DNA-protein interactions.

Another DNA-binding protein (DBP) related contribution by Yu et al. shows the development of a novel predictor called Hybrid_DBP for identifying DNA-binding proteins (DBP) using hybrid features and convolutional neural networks. The study highlighted the importance of studying DBP for understanding genetics, evolution, and disease prevention and treatment. The Hybrid_DBP predictor combines two feature selection methods, MonoDiKGap and Kmer, and uses MRMD2.0 to remove redundant features. The results show that Hybrid_DBP correctly recognizes 94% of DBP and achieves an accuracy of 91.2% on an independent test set.

Drug-drug interaction (DDI) studies examine how a drug’s effects can change when it is administered concurrently with another medication. Lin et al. proposed a novel method of multiview fusion based on dual-level attention for drug interaction prediction. The current methods for DDI prediction have limitations in integrating drug features and network structures, considering only a single view of drug interaction and ignoring the importance of different neighbors. To address these challenges, The proposed method constructs multiple views of the drug interaction relationship and adopts a cross-fusion strategy to fuse drug features with the drug interaction network under each view. It also incorporates a dual-level attention mechanism to distinguish the importance of different neighbors and views. In the multi-task analysis of new drug reactions, the proposed method achieved superior scores across various metrics. Furthermore, its predictive outcomes were consistent with particular drug reaction events, leading to more precise predictions.

Ai et al. looked at a very specific DDI Research Topic, the activity prediction of cytochrome P450(CYP). CYPs are enzymes involved in the metabolism of drugs, xenobiotics, and endogenous compounds, and their inhibition can lead to adverse drug-drug interactions. In this study, the Fingerprint-Graph Neural Network (FP-GNN) learning method is used to construct classification models for predicting CYP inhibition. The evaluation results showed that the multi-task FP-GNN model achieved the best predictive performance compared to other machine learning and deep learning models. The model’s interpretability allowed for the identification of critical structural fragments associated with CYP inhibition. An online application called DEEPCYPs was created based on the optimal multi-task FP-GNN model to detect potential CYP inhibitors. The FP-GNN model has been reported to achieve high accuracy on various datasets and has been successfully applied in predicting inhibitors of different enzymes and evaluating the anti-cancer activity of compounds.

Biomedical named entity recognition (BioNER) is a task that aims to automatically extract and classify named entities within large volumes of biomedical literature, research papers, clinical notes, and other texts. As natural language processing technology advances, a plethora of deep learning models are being employed to extract valuable insights from biomedical literature. This advancement contributes to the growth of efficient BioNER models. Han et al. developed a method that integrates BERT-processed text embeddings and entity dependencies using a graph attention network (GAT) to construct better entity embedding representations. The results show that the method outperforms existing methods on three datasets, NCBI-disease corpus, BC2GM, and BC5CDR-chem, achieving competitive accuracy and higher efficiency. The study concludes that incorporating drug, disease, and protein dependencies can enhance the representation of entities in neural networks and improve BioNER performance. The research findings suggest that incorporating drug, disease, and protein dependencies can improve the representation of entities in the neural network model and, consequently, enhance the performance of BioNER.

Lesion studies can play a role in drug development. In many diseases, lesions are key diagnostic indicators. Imaging techniques like MRI, CT scans, and PET scans can help detect lesions in the body, including the brain, and lungs (e.g., tumours and nodules). These imaging techniques are crucial for identifying individuals who could potentially benefit from a particular drug or therapeutic strategy. Wang et al. present a method for universal lesion detection (ULD) based on partially supervised learning (PSL). The authors propose a novel loss function that includes a negative sample proportion reduction factor and a masking strategy to address the imbalance Research Topic of anchors in lesion detection tasks. The method generates a mask that intentionally chooses fewer negative anchors and sets a parameter to reduce the proportion of negative samples, thereby improving the performance of the lesion detection model. The experiments are conducted on a large-scale public dataset called DeepLesion. The proposed method significantly improves the performance of the ULD detector.

## 4 Conclusion

Following the publication of the special thematic as a “Research Topic” on the Frontiers in Pharmacology journal website, it attracted an immense amount of attention and interest from researchers in the drug design and development field. After a careful and rigorous review and selection process, seven contributions were eventually published in this Research Topic. The acceptance rate is about 40%. The encompasses a wide range of studies within the Research Topic. The seven contributions include one paper pertaining to DTI, two related to PPI, two discussing DDI, one concerning BioNER, and one focused on lesion detection. At the time of writing this editorial, the Research Topic has received 7,963 total views, 6,338 article views and 1,836 downloads. We sincerely express our gratitude to Frontiers in Pharmacology team for granting us the opportunity to curate this Research Topic. We extend special thanks and appreciation to all the contributors for their exceptional work and to the anonymous reviewers who generously dedicated their time and expertise to assess and comment on the submitted manuscripts. Without their invaluable contributions, this Research Topic could not have achieved such success.
